# Longitudinal Study of Functional Reinnervation of the Denervated Skin by Collateral Sprouting of Peptidergic Nociceptive Nerves Utilizing Laser Doppler Imaging

**DOI:** 10.3389/fphys.2020.00439

**Published:** 2020-05-21

**Authors:** Szandra Lakatos, Gábor Jancsó, Ágnes Horváth, Ildikó Dobos, Péter Sántha

**Affiliations:** ^1^Department of Physiology, University of Szeged, Szeged, Hungary; ^2^1st Department of Internal Medicine, University of Szeged, Szeged, Hungary

**Keywords:** sensory innervation, nociception, cutaneous vasodilatation, nerve injury, collateral sprouting, scanning laser Doppler flowmetry, TRPV1, TRPA1

## Abstract

Restitution of cutaneous sensory function is accomplished by neural regenerative processes of distinct mechanisms following peripheral nerve lesions. Although methods available for the study of functional cutaneous nerve regeneration are specific and accurate, they are unsuitable for the longitudinal follow-up of the temporal and spatial aspects of the reinnervation process. Therefore, the aim of this study was to develop a new, non-invasive approach for the longitudinal examination of cutaneous nerve regeneration utilizing the determination of changes in the sensory neurogenic vasodilatatory response, a salient feature of calcitonin gene-related peptide-containing nociceptive afferent nerves, with scanning laser Doppler flowmetry. Scanning laser Doppler imaging was applied to measure the intensity and spatial extent of sensory neurogenic vasodilatation elicited by the application of mustard oil onto the dorsal skin of the rat hindpaw. Mustard oil induced reproducible and uniform increases in skin perfusion reaching maximum values at 2–4 min after application whereafter the blood flow gradually returned to control level after about 8–10 min. Transection and ligation of the saphenous nerve largely eliminated the vasodilatatory response in the medial aspect of the dorsal skin of the hindpaw. In the 2^*nd*^ to 4^*th*^ weeks after injury, the mustard oil-induced vasodilatatory reaction gradually recovered. Since regeneration of the saphenous nerve was prevented, the recovery of the vasodilatatory response may be accounted for by the collateral sprouting of neighboring intact sciatic afferent nerve fibers. This was supported by the elimination of the vasodilatatory response in both the saphenous and sciatic innervation territories following local treatment of the sciatic nerve with capsaicin to defunctionalize nociceptive afferent fibers. The present findings demonstrate that this novel technique utilizing scanning laser Doppler flowmetry to quantitatively measure cutaneous sensory neurogenic vasodilatation, a vascular response mediated by peptidergic nociceptive nerves, is a reliable non-invasive approach for the longitudinal study of nerve regeneration in the skin.

## Introduction

Lesions of peripheral nerves are often inflicted by common traumatic injuries or toxic agents of environmental and medicinal origins resulting in axonal degeneration and consequent loss of neural function. Restitution of cutaneous sensory functions may ensue rapidly after nerve injury. The return of sensation may be studied with various methods including measurements of changes in the thresholds to nociceptive mechanical and thermal stimuli or histological examination of skin biopsy specimens (Nolano et al., 1999; Kennedy et al., 2010; Cobianchi et al., 2014). Alternatively, reinnervation of denervated skin territories may be evaluated with the Evans blue technique by demonstrating skin areas of increased vascular permeability induced by antidromic electrical or direct chemical stimulation of nociceptive nerve endings (Jancsó and Király, 1983; Bharali and Lisney, 1988; Brenan et al., 1988; Lisney, 1989). Although these techniques are specific and accurate in determining skin areas innervated by nociceptive afferents, they have significant limitations. Techniques utilizing measurements of nociceptive thresholds and evaluation of biopsy specimens are unsuitable for the exact delineation of innervated and denervated skin regions. In contrast, the Evans blue method exactly outlines the innervated skin areas, but similarly to the biopsy technique, its use is limited in longitudinal studies.

Peptidergic chemosensitive primary sensory neurons which express the nociceptive ion channels transient receptor potential vanilloid type 1 (TRPV1) and transient receptor potential ankyrin type 1 (TRPA1) comprise a unique population of sensory neurons with dual nociceptive and secretory functions. Besides the transmission of nociceptive stimuli toward the central nervous system, chemosensitive sensory nerve endings through the release of neuropeptides such as substance P and calcitonin gene-related peptide are involved in mediation of local tissue reactions, including vascular changes (Jancsó et al., 1987; Maggi et al., 1987; Maggi and Meli, 1988; Holzer, 1998; Geppetti et al., 2008; Jancsó et al., 2009). Electrophysiological studies also disclosed that vasodilator afferent nerves are nociceptive C-fibers mostly comprised of nociceptor polymodal units in the rat (Gee et al., 1997). Ample experimental evidence indicates that sensory neurogenic plasma extravasation and sensory neurogenic vasodilatation are elicited by substance P and calcitonin gene-related peptide, respectively, released from activated chemosensitive afferent nerves (Lembeck and Holzer, 1979; Gamse et al., 1980; Brain et al., 1985; Holzer, 1998; Boros et al., 2016). Cutaneous sensory neurogenic vasodilatation can be reliably demonstrated and measured utilizing measurement of skin blood flow with laser Doppler flowmetry (Lynn et al., 1996; Gee et al., 1997).

Scanning laser Doppler flowmetry is a reliable and useful technique for the measurement of cutaneous blood flow under physiological and pathophysiological conditions including thermoregulatory vascular responses in the intact and denervated skin in both man and animals (Hu et al., 2012; Deng et al., 2016). Laser Doppler flowmetry directly measures the cutaneous blood perfusion based on the detection of frequency (“Doppler”) shift of low-power monochromatic laser light reflected from the moving erythrocytes, but not from the stationary tissue elements in the skin (Rajan et al., 2009; Daly and Leahy, 2013; Allen and Howell, 2014). The magnitude of the intensity of shifted reflected light is proportional to the local concentration of moving erythrocytes, whereas the mean of frequency change is proportional to the average velocity of moving red blood cells (Fredriksson et al., 2009; Rajan et al., 2009). Usually an integrated value reflecting simultaneously the changes in red blood cell concentration and velocity is calculated and the tissue perfusion is expressed as an arbitrary perfusion unit (Rajan et al., 2009; Daly and Leahy, 2013). Typical laser Doppler flowmetry assesses blood flow in the microcirculation of the superficial approximately 1 mm thick layer of the skin (Fredriksson et al., 2009). Laser Doppler perfusion imaging is also based on this principle, however a point-to-point sampling on a pre-defined skin area is performed by using scanning mirrors and computerized control and processing system. The recorded perfusion map shows the local distribution of skin areas exhibiting different blood perfusion intensities. The spatial resolution of the perfusion imaging is influenced by numerous factors, but the shortest distance which could be resolved is close to the 0.1 mm range (Rajan et al., 2009; Allen and Howell, 2014). This technique is suitable not only for the measurement of changes in skin blood flow but also to determine the topographical distribution of the changes in skin perfusion. Examination of cutaneous vasodilatatory responses elicited through orthodromic chemical stimulation of afferent nerve endings is a reliable technique for the demonstration of the functional innervation of the skin and the identification of innervated and denervated skin areas (Domoki et al., 2003; Illigens et al., 2013). The present experiments were initiated in an attempt to evaluate and validate scanning laser Doppler imaging as a novel non-invasive approach for the longitudinal study of the functional regeneration of cutaneous nociceptive nerves.

To support the experimental findings on degeneration and regeneration of cutaneous sensory nerves as assessed with the technique of scanning laser Doppler flowmetry, further experiments were performed using immunohistochemistry. The method of vascular labeling was utilized to identify innervated and denervated skin areas following transection of the saphenous nerve. This technique is based on the visualization of colloidal silver deposited in the basal membrane of permeable small blood vessels, mostly postcapillary venules following the epicutaneous application of mustard oil to induce neurogenic inflammation (Jancsó, 1960; Jancsó et al., 1980a). Neurogenic inflammation is a collective term for neurogenic sensory vasodilatation, mediated primarily by CGRP, and neurogenic plasma extravasation, mediated by substance P upon orthodromic or antidromic stimulation of sensory nerves (Jancsó, 1960; Jancsó et al., 1967, 2009; Lembeck and Holzer, 1979; Brain et al., 1985; Maggi and Meli, 1988; Holzer, 1998). Vascular labeling is a salient feature of increased vascular permeability (Jancsó, 1955; Majno et al., 1961). Detection of vascular labeling is a reliable measure to gather information on the functional state of sensory nerves mediating these vascular reactions, since CGRP is co-localized in almost all substance P-containing nerve fibers (Ju et al., 1987). The permeable blood vessels can be easily identified under the light microscope by the presence of colloidal silver in their walls (Dux and Jancsó, 1994). Neurogenic inflammation and vascular labeling cannot be induced in the denervated skin (Jancsó, 1955, 1960; Jancsó et al., 1967, 1980a, 1987; Pertovaara, 1988; Sann et al., 1995; Dux et al., 1998).

## Materials and Methods

### Animals

In total of eight adult male Wistar rats weighing 250–280 g at the beginning of the experiments were used in this study. The animals were maintained under a 12-h light/dark cycle with free access to food and water. The experiments were approved by the Ethics Committee for Animal Care at the University of Szeged as per the Council Regulation of 40/2013 (II. 14.) and were carried out in full accordance with the European Communities Council Directive of 24 November 1986 (86/609/EEC). All efforts were made to minimize animal suffering. The number of experimental animals was kept as low as possible.

### Surgery

For surgical interventions rats were anesthetized with a combination of ketamine (Calypsol, 70 mg/kg, i.p., Gedeon Richter, Budapest, Hungary) and xylazine (CP-Xylazin 2%, 10 mg/kg, i.p., Produlab Pharma, Raamsdonksveer, Netherlands).

### Peripheral Nerve Transection

The right saphenous nerve was exposed high in the thigh and transected distal to a ligature. To prevent regeneration of the nerve, a 0.5 cm long segment of the distal stump was removed. The wound was then closed and the rat was returned to the animal house.

### Perineural Capsaicin Treatment

Perineural application of capsaicin was performed as described by Jancsó et al. (1980b). Briefly, the right saphenous nerve was exposed high in the thigh, isolated from the surrounding tissues with Parafilm^®^ (Sigma-Aldrich) and wrapped with a small piece of gelfoam soaked with 0.1 ml of a 1% solution of capsaicin (Sigma, Saint Louis, United States) dissolved in saline containing 6% ethanol and 8% Tween 80. After 20 min, the gelfoam was removed, the wound was closed, and the rat was returned to the animal house.

### Measurement of Cutaneous Blood Flow With Scanning Laser Doppler Flowmetry

Scanning laser Doppler flowmetry was used to measure cutaneous blood flow in the dorsal skin of the rat hindpaw by capturing consecutive perfusion images with a PeriScan PIM3 scanning laser Doppler imager (Perimed, Järfälla, Sweden). The rats were anesthetized with a combination of ketamine and xylazine and then were placed on a heating pad to keep their body temperature at 37 ± 0.5°C. Room temperature was kept at 22–23°C. The dorsal surface of both hindpaws was scanned by using the repeated scan mode with 52 × 42 pixel frame size. Distance of the scanner aperture from the skin surface was set to 19 cm and the scanner was positioned to ensure that the laser beam was perpendicular to the skin surface. Perfusion images were captured in every 2 min and measurements took 15–20 min in each animal. All flow values were expressed as means ± S.E.M. Basal tissue perfusion and changes in blood flow induced by mustard oil (5% in liquid paraffin) were recorded in arbitrary perfusion units (PU) and expressed as per cent change relative to baseline. The value of the PU integrates the linear velocity values and the concentration of moving erythrocytes in the skin volume fraction detected by the scanner at any instances (Rajan et al., 2009). Mustard oil was applied onto the intact unshaved hairy skin of the paw. Baseline values were obtained by calculating the average of three subsequent measurements before the application of mustard oil. For quantitative evaluation, images displaying the maximum vasodilatatory responses were used in each experiment.

Scanning laser Doppler images were taken before surgery and 1–40 days after saphenous nerve transection. After finishing the sequential measurement of the recovery of sensory vasodilatation (4th week post surgery) an additional measurement was made 4 days after perineural treatment of the sciatic nerve with capsaicin.

The innervation territory of the saphenous nerve was defined on the basis of perfusion images taken 4 days after transection the saphenous nerve. In each experiment the color-coded perfusion images showing the maximal vasodilatatory response were selected for further processing with the ImagePro 6.2 image analysis software (MediaCybernetics, Rockville, MD, United States). After subtracting background pixel values, color segmentation was applied on the perfusion image to demarcate and separate areas showing no or minimal vasodilation from those exhibiting large (or maximal) perfusion increases. This step was followed by the generation of a binary mask representing the size and topography of denervated cutaneous areas corresponding to the innervation area of the saphenous nerve. Functional reinnervation was characterized by measuring the intensity of the vasodilatatory response in the saphenous skin area as defined above.

### Demonstration of Cutaneous Nerve Fibers and Permeable Blood Vessels After Induction of Neurogenic Inflammation With Mustard Oil

Rats were anesthetized with ketamine (100 mg/kg) and xylazine (10 mg/kg) and the dorsal skin of the hind paw was painted with mustard oil (allyl isothiocyanate, 5% in liquid paraffin) after an intravenous injection of a colloidal silver solution (1% in 5% glucose, 100 mg/kg). Twenty min later the animals were terminally anesthetized and perfused transcardially with a fixative containing 4% paraformaldehyde in 0.1 M phosphate buffer. The dorsal hind paw skin was removed and postfixed for another 2 h. After washing in phosphate buffer containing 30% sucrose overnight, frozen sections were cut and free-floating sections were processed for immunohistochemical staining with antibodies against tubulin and CGRP. Monoclonal mouse anti-ß-tubulin III and polyclonal rabbit anti-CGRP antibodies were obtained from Sigma-Aldrich (St. Louis, Missouri, United States) and used at a dilution of 1:4500. Sections were mounted on slides and covered with ProLong^®^ Gold antifade medium (Invitrogen, Carlsbad, Calif., United States). Specimens were examined under a Zeiss LSM 700 confocal laser scanning microscope. Tile scan and z-stack maximum intensity projection images were obtained to illustrate the findings.

### Statistics

Data represent means ± S.E.M. of 5–9 independent measurements. For statistical comparisons of the mustard oil-induced vasodilatatory responses one-way ANOVA was performed followed by multiple comparisons using the Dunett’s *post hoc* analysis. In all groups, normality was proved by the Shapiro-Wilk test and homogeneity of variances was confirmed by Levene’s test in advance of performing ANOVA. Statistical analysis was performed by using Statistica 6.4 software (Dell Inc., Tulsa, OK, United States).

## Results

### Scanning Laser Doppler Imaging of Cutaneous Blood Flow of the Dorsal Skin of the Rat Hindpaw: The Effect of Sensory Denervation

**FIGURE 1:**
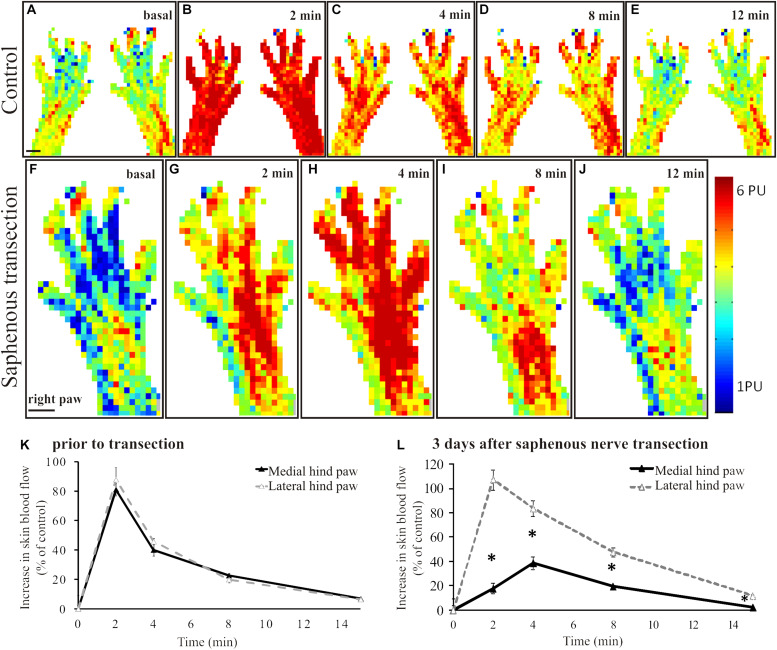
**A–E**: Original perfusion images recorded with the scanning laser Doppler flowmeter illustrating the time course and spatial distribution of the vasodilatatory response elicited by the epicutaneous application of mustard oil onto the dorsal skin of the hindpaws of a control rat. Note the increase in blood flow of similar magnitude in the medial and lateral regions of the hindpaw skin. The line graph (**K**) depicts the time course of the increase in cutaneous blood flow relative to baseline value. Filled and empty triangles represent values measured in the medial (saphenous innervated) and lateral (sciatic innervated) skin areas, respectively (*n* = 6). **F–J:** Original perfusion images recorded with the scanning laser Doppler flowmeter illustrating the reduced mustard oil-induced vasodilatation in the medial aspect of the dorsal hindpaw skin three days after transection of the saphenous nerve. The line graph (**L**) shows the time course of the vasodilatatory response in the medial (filled triangles) and lateral (empty triangles) aspects of the dorsal hindpaw skin (*p < 0.05; *n* = 6). Scale bar on A and F represents 5 mm.

Scanning laser Doppler imaging revealed a largely uniform perfusion in the intact rat hindpaw skin (Figure 1A). The coefficients of variance of the basal perfusion values recorded on subsequent perfusion images representing the basal blood perfusion of the hind paw were 0.043 ± 0.28 (medial aspect) and 0.054 ± 0.027 (lateral aspect) with no significant difference between the two sides (*p* = 0.19). Application of mustard oil onto the skin elicited a marked increase in cutaneous blood flow with a maximum at 2–4 min (Figures 1B,K). The maximum increase in blood flow amounted to 87 ± 18% of the initial basal value and returned to the pre-application level after about 15–20 min (Figures 1C–E,K). This vasodilatatory response could be repeatedly elicited by mustard oil resulting in similar perfusion patterns (data not shown). Basal blood flow measured in the denervated (saphenous) skin area was similar to that of the intact lateral (sciatic) area of the dorsal skin of the hindpaw (Figure 1F). However the mustard oil-induced vasodilatatory response was strongly reduced in the medial aspect of the dorsal hindpaw skin 3 days after saphenous nerve denervation (Figures 1G,H decline of the response is shown on Figures 1I,J). As illustrated in Figure 1L, the magnitude of the vasodilatatory response was reduced significantly in the medial aspect of the dorsal hindpaw skin served by the saphenous nerve, as compared with the lateral side innervated by the intact sciatic nerve.

### Effect of Saphenous Nerve Transection on Mustard Oil-Induced Vasodilation in the Dorsal Hindpaw Skin

**FIGURE 2:**
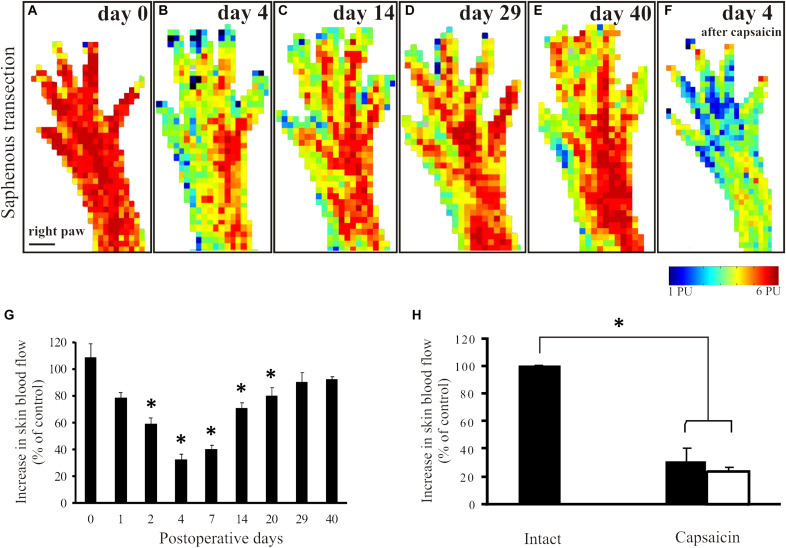
Mustard oil-induced changes in blood flow of the dorsal hindpaw skin after transection and ligation of the saphenous nerve. Original perfusion images demonstrate mustard oil-induced vasodilatatory responses before (**A**), and 4 (**B**), 14 (**C**), 29 (**D**), and 40 (**E**) days after surgery. Note the gradual recovery of the vasodilatatory response in the medial aspect of the hindpaw. Image **F** illustrates the effect of the selective defunctionalization by perineural capsaicin treatment of sciatic afferents on the mustard oil-induced vasodilation. Note the marked reduction of the vasodilatatory response in both the lateral and the medial aspects of the dorsal hindpaw skin served by the sciatic and saphenous nerves, respectively, after capsaicin treatment. **G:** The histogram shows the quantitative data on changes in the vasodilatatory responses following saphenous nerve transection. Note the marked decrease shortly after transection and the gradual recovery over a period of 4–5 weeks, indicating denervation and reinnervation of the denervated saphenous skin area. **H:** Forty days after saphenous nerve transection and four days after perineural capsaicin treatment of the sciatic nerve significant reductions of mustard oil-induced vasodilatation in both the medial (filled column) and lateral (empty column) aspect of the dorsal hindpaw skin were detected as compared to the vasodilatatory response measured in the contralateral dorsal hindpaw skin (^∗^*p* < 0.05; *n* = 8). Scale bar on A represents 5 mm.

In animals to be subjected to transection and ligation of the saphenous nerve, mustard oil-induced vasodilatatory responses were measured shortly before surgery to obtain reference perfusion images (Figure 2A). Basal blood flow values did not differ significantly in the denervated and innervated areas of the dorsal hindpaw skin (Figure 1F). Four days but not one day after saphenous nerve transection mustard oil-induced increase in cutaneous blood flow was markedly reduced in the medial part of the dorsal hindpaw skin served by the saphenous nerve. Perfusion values in the skin area normally served by the saphenous nerve amounted to about 32 ± 4% of the values measured in the lateral hindpaw skin innervated by the intact sciatic nerve (Figures 2B,G). The vasodilatatory response gradually recovered within the medial skin area of the dorsal hindpaw. The re-appearance of mustard oil-induced vasodilation first commenced in the lateral aspect of the saphenous innervation territory immediately adjacent to the skin area innervated by the intact sciatic nerve (Figures 2C,D). The area displaying mustard oil-induced vasodilation gradually spread toward the medial aspect of the dorsal hindpaw skin and finally, after about 4 weeks the topography of the vasodilatatory response was similar to that seen before surgery (Figure 2E). To identify the origin of nerve fibers which reinnervated the medial part of the dorsal hindpaw skin served normally by the saphenous nerve, a second surgery was performed. The sciatic nerve was treated locally with capsaicin to defunctionalize chemosensitive afferent nerves (Jancsó et al., 1980b; Gamse et al., 1980) which mediate the sensory neurogenic vasodilatatory response (Lembeck and Holzer, 1979; Lynn et al., 1996; Domoki et al., 2003). Examination of the mustard oil-induced vasodilatatory response 3–4 days after perineural capsaicin revealed a marked reduction of the vasodilatatory response not only in the lateral skin area, innervated by the sciatic nerve, but also in the medial skin area normally served by the saphenous nerve (Figures 2F,H). This finding strongly indicates that recovery of the vasodilatatory response in the medial part of the dorsal hindpaw skin, normally served by the saphenous nerve, may be attributed to sciatic afferents which innervate the denervated saphenous skin area through collateral sprouting.

### Immunohistochemical Demonstration of Degeneration and Regeneration of Cutaneous Nerves After Peripheral Nerve Lesions

**FIGURE 3:**
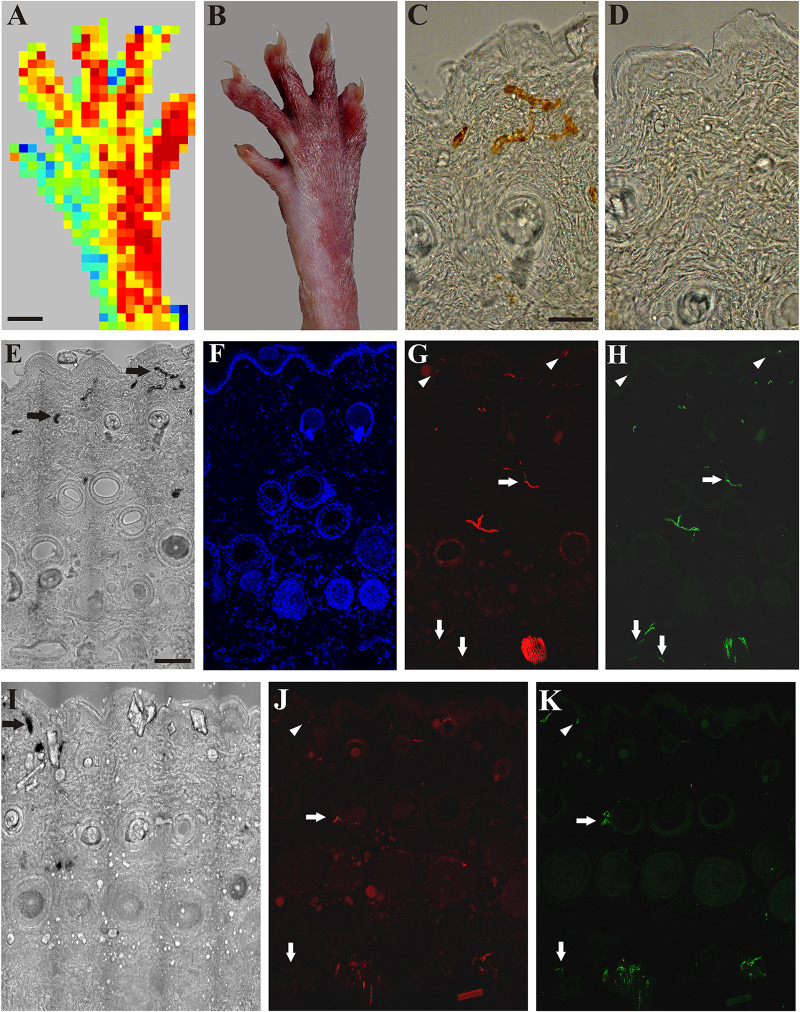
**A:** Application of mustard oil increased skin blood flow in the innervated (sciatic) but not in the denervated (saphenous) skin area of the right hind paw as assessed with scanning laser Doppler imaging. **B:** Brownish coloration of the skin due to silver accumulation in the innervated (sciatic) but not in denervated (saphenous) skin area of the dorsal hind paw skin elicited by mustard oil. **C, D**: Bright field photomicrographs showing sections from the lateral intact (**C**) and medial denervated regions of the hind paw skin after transection of the saphenous nerve (**D**). Silver-labeled small venules indicate increased vascular permeability of small postcapillary venules elicited by mustard oil application in the intact dorsal hind paw skin (**C**). Silver-labeled blood vessels cannot be observed in the denervated skin 4 days after saphenous nerve transection (**D**). **E–H**: Bright field (**E**) and immunofluorescence (**F–H**) photomicrographs showing the lateral (sciatic) skin area of the dorsal hind paw skin (**E–H**) after transection of the saphenous nerve. **I–K**: Bright field (**I**) and immunofluorescence (**J, K**) photomicrographs showing the medial (saphenous) skin area of the dorsal hind paw skin of a rat 15 days after transection and ligation of the saphenous nerve. Note the localization of ß-tubulin III (red) and CGRP-immunoreactive (green) nerve fibers in the epidermis (arrowheads), and around hair follicles and small arteries (arrows) in the innervated (lateral, sciatic) area of the dorsal hind paw skin identified by the presence of silver-labeled (permeable) venules (arrows in **E**). Fifteen days after transection and ligation of the saphenous nerve, some silver-labeled venules (arrow in **I**) and some ß-tubulin III and CGRP-immunoreactive epidermal (arrowheads in **J, K**) and dermal (arrows in **J, K**) nerve fibers can be observed in the medial (saphenous) skin area of the dorsal hind paw skin indicative of (collateral) regeneration. Scale bars indicate 5 mm in **A** and **B** and 50 μm in **C** and **E**. Scale bars in **C** and **E** apply for **C, D** and **E–K**, respectively.

Application of mustard oil resulted in clear-cut vascular labeling in subepidermal small blood vessels of skin areas of intact sensory innervation (perfusion image: Figure 3A). Silver deposits were observed in the wall of permeable small blood vessels (Figures 3B,C). In contrast, in the denervated skin vascular labeling could not be observed (Figures 3B,D). Immunohistochemistry revealed many tubulin- and/or CGRP-immunoreactive nerve fibers in the epidermis, around hair follicles and small blood vessels of the innervated skin (Figures 3E–H). Four days after nerve transection, nerve fibers were absent in the denervated saphenous skin areas, but the innervation of the skin area served by the intact sciatic nerve was similar to control (data are not shown). Fourteen days after saphenous nerve transection, a few silver-labeled blood vessels could be detected in the previously denervated skin area parallel with the re-appearance of a few epidermal and subepidermal tubulin- and CGRP-immunoreactive nerve fibers indicating regeneration of the denervated skin area (Figures 3I–K). These nerves were completely depleted after perineural treatment of the sciatic nerve with capsaicin. Hence, these immunohistochemical findings extend and support our observations on the regeneration of the denervated skin as assessed with the aid of scanning laser Doppler flowmetry.

## Discussion

Longitudinal evaluation of restitution of cutaneous sensory function is essential to unravel the progress and mechanisms of nerve regeneration following peripheral nerve lesions of various origins. The findings of the present study demonstrate that repeated scanning laser Doppler imaging of cutaneous blood flow is a reliable method for the longitudinal examination of the progress of regeneration of a particular population of peptidergic nociceptive cutaneous nerves. Stimulation of cutaneous nociceptive nerve endings with mustard oil results in a marked increase of local blood flow in intact but not denervated skin areas. Previous studies have shown that mustard oil is a potent activator of the TRPA1 nociceptive ion channel (Jordt et al., 2004; Bautista et al., 2006). Although mustard oil was regarded as a selective agonist of the TRPA1 receptor, recent findings challenged this view by showing activation also of the TRPV1 receptor by this compound suggesting that besides the TRPA1 receptor, the TRPV1 receptor is also involved in the transmission of nociceptive impulses elicited by mustard oil (Everaerts et al., 2011; Gees et al., 2013). However, these studies also disclosed that mustard oil-induced inflammation is mainly mediated by the TRPA1 receptor (Everaerts et al., 2011; Gees et al., 2013). This is in accord with our previous observations showing that the vasodilatatory effect of this agent is largely mediated by the activation of the TRPA1 receptor in the rat hindpaw skin (Boros et al., 2016). The vasodilation elicited by activation of the TRPA1 receptor by mustard oil is mediated by the potent vasodilatatory peptide CGRP contained and released from the stimulated nociceptive nerve endings (Brain et al., 1985; Sauerstein et al., 2000; Pozsgai et al., 2010). This notion is also supported by the close spatial correlation of the distribution of CGRP-containing chemosensitive afferent nerves and vascular changes associated with neurogenic inflammation in the rat skin (Sann et al., 1995).

Denervated skin areas displayed markedly attenuated vasodilatatory responses upon exposure to mustard oil. The slight mustard oil-induced increase in blood flow measured in the denervated skin areas may be attributed to possible non-neural mechanism(s) or, alternatively, to activation of TRPV1 receptors (Grant et al., 2005; Everaerts et al., 2011; Gees et al., 2013).

Mustard oil-induced vasodilation was markedly reduced in the denervated skin 3–4 days after nerve transection and was abolished completely by the 4th post-operative day. The vasodilatatory response was similar to controls 1 day after nerve transection, since at this post-lesion time cutaneous nerve endings are still functional until the onset of the rapid phase of Wallerian degeneration 1–3 days after injury (Jancsó and Király, 1983; Sta et al., 2014). Repeated measurements of mustard oil-induced vasodilatatory responses revealed a gradual, time-dependent re-appearance of the vasodilatatory response in the denervated skin. Following saphenous nerve transection, the size of the skin area displaying negligable vasodilatatory responses after denervation gradually decreased. By the 3rd-4th post-operative week, the vasodilatatory response was similar to the control in both size and intensity. To furnish independent data on the reliability of scanning laser Doppler flowmetry to examine cutaneous nerve function, immunohistochemical demonstration of tubulin- and CGRP-positive nerves were performed in intact, denervated and reinnervated skin after induction of neurogenic inflammation with mustard oil. In accord with previous findings, small blood vessels, mainly postcapillary venules were labeled with colloidal silver. No such labeled venules were seen in the denervated skin. Re-appearance of tubulin- and CGRP-immunoreactive nerve fibers in the previously denervated saphenous skin area supported our findings obtained with scanning laser Doppler flowmetry. The nerve fibers in the saphenous skin area were completely depleted after perineural treatment of the sciatic nerve with capsaicin, a treatment which induces a complete elimination of nociceptive afferent nerves (Jancsó et al., 1980b; Dux et al., 1998; Domoki et al., 2003; Kang et al., 2010). Hence, immunohistochemical findings supported our observations obtained with scanning laser Doppler flowmetry. This time-course of functional recovery observed in the previously denervated saphenous innervation area is similar to that observed in studies using behavioral testing or the Evans blue technique to examine the temporal characteristics of functional cutaneous nerve regeneration (Devor et al., 1979; Bharali and Lisney, 1988).

Previous studies have demonstrated that reinnervation of denervated skin areas may be effected by two different mechanisms: regeneration of the injured nerve and reinnervation by collateral sprouting (Devor et al., 1979; Brenan, 1986; Diamond et al., 1987; Kinnman et al., 1992). The re-innervation of the denervated skin following nerve crush occurs through regenerative sprouting of the injured nerve. The restitution of function is almost complete after nerve crush (Devor et al., 1979; Wiesenfeld-Hallin et al., 1988). If regeneration of the injured nerve is prevented by ligation of the transected nerve, as in the present study, restitution of sensory function in the denervated skin is accomplished through collateral sprouting of axons of an intact peripheral nerve serving skin areas adjacent to the denervated skin (Devor et al., 1979; Diamond et al., 1987; Pertovaara, 1988). Under the experimental conditions of the present study, intact sciatic nerve axons were expected to invade, by collateral sprouting, the denervated saphenous skin area. Our findings indicate that, indeed, this is the case. In the denervated saphenous nerve territory, the mustard oil-induced vasodilatatory response gradually recovered within a period of 1-4 weeks after transection and ligation of the saphenous nerve and, importantly, this could be largely abolished by perineural treatment of the sciatic nerve with capsaicin. Perineural capsaicin treatment was used to selectively defunctionalize sciatic nociceptive afferents (Jancsó et al., 1980a; Jancsó et al., 2008; Jancsó and Király, 1983; Petsche et al., 1983; Pini and Lynn, 1991; Domoki et al., 2003) sparing efferent autonomic and motor nerve fibers (Jancsó et al., 1987). Thus, inhibition by perineural capsaicin treatment of the mustard oil induced vasodilation not only in the lateral, but also in the medial aspect of the dorsal skin of the hindpaw, strongly indicates that reinnervation of the denervated saphenous skin area was accomplished by collateral sprouting of adjacent intact sciatic afferent fibers. This observation is in accord with previous findings which applied the Evans blue technique and behavioral testing to demonstrate the reinnervation of the denervated skin by collateral sprouting of nociceptive afferent nerves of neighboring skin areas (Devor et al., 1979; Diamond et al., 1987; Pertovaara, 1988).

In conclusion, the present observations indicate that repeated scanning laser Doppler imaging of the mustard oil-induced vasodilatatory response is a reliable technique for the longitudinal study of cutaneous nerve regeneration being suitable for the follow-up of changes of both the topographical distribution and the intensity of the vasodilatatory response, most probably proportional with cutaneous innervation density. An obvious limitation of this technique is that it does not provide information on the regeneration of other types of cutaneous sensory nerves, for example myelinated mechanoreceptors. Noteworthy, it has been shown that low threshold myelinated afferents lack the ability to collaterally grow into a denervated skin area (Jackson and Diamond, 1984). In addition, however, previous findings suggested that regeneration of different types of cutaneous nerves does not occur simultaneously; unmyelinated fibers regenerate more rapidly as compared to myelinated axons (Allt, 1976; Duraku et al., 2013). Human and rodent nociceptive cutaneous nerves share many functional and neurochemical traits, including the sensitivity to chemical irritants such as mustard oil and capsaicin (Jancsó, 1960; Jancsó et al., 1985; Hou et al., 2002). In the human skin mustard oil produces local and axon reflex vasodilation in the intact but not in the denervated skin (Jancsó and Janka, 1981; Jancsó et al., 1983, 1985). Demonstration of mustard oil-induced sensory neurogenic vasodilation has been used to detect the functional condition of cutaneous sensory nerves in man under physiological and pathological conditions (Jancsó and Janka, 1981; Jancsó et al., 1983; Westerman et al., 1987). Hence, with some modifications, the approach presented in this report may be applied also in human studies aimed at the examination of cutaneous nerve function affected by toxic environmental and medicinal agents, such as anticancer chemoterapeutics or by pathologies such as diabetes mellitus.

## Ethics Statement

The animal study was reviewed and approved by the Ethics Committee for Animal Care at the University of Szeged as per the Council Regulation of 40/2013 (II. 14.) and were carried out in full accordance with the European Communities Council Directive of 24 November 1986 (86/609/EEC).

## Data Availability Statement

The data that support the findings of this study are available on request from the corresponding author, PS.

## Author Contributions

GJ, PS, and SL were responsible for the study concept and design. SL, ÁH, ID, GJ and PS were responsible for the data collection and analysis. SL, ÁH, GJ, and PS interpreted the data, and revised the manuscript for intellectual content. All authors were involved in manuscript editing and have approved the final version for submission.

## Conflict of Interest

The authors declare that the research was conducted in the absence of any commercial or financial relationships that could be construed as a potential conflict of interest.

## Abbreviations

CGRP: calcitonin gene-related peptide
PU: perfusion unit
TRPA1: transient receptor potential ankyrin type 1 receptor
TRPV1: transient receptor potential vanilloid type 1 receptor.
